# Cystic brain metastases in ALK-rearranged non-small cell lung cancer

**DOI:** 10.3332/ecancer.2018.818

**Published:** 2018-03-14

**Authors:** Guilherme Nader Marta, Renata Rodrigues da Cunha Colombo Bonadio, Renata Eiras Martins, Henrique Bortot Zuppani, Gilberto de Castro

**Affiliations:** Instituto do Câncer do Estado de São Paulo, São Paulo, SP 01246-000, Brazil

**Keywords:** non-small cell lung cancer, anaplastic lymphoma kinase, cystic brain metastases, crizotinib, neurocysticercosis

## Abstract

The central nervous system (CNS) is a common site of disease progression in patients with non-small-cell lung cancer (NSCLC) with anaplastic lymphoma kinase (ALK)-rearrangement treated with crizotinib. Cystic brain metastases (CBM) have been recently identified as one possible variant of this disease. An illustrative case report is presented along with a literature review performed in order to track relevant papers about CBM in ALK-rearranged NSCLC, including possible pathophysiology, differential diagnosis and treatment options for this condition. Three case reports have been published describing six ALK-rearranged NSCLC patients presenting with CBM, all of which were under treatment with crizotinib by the time of CBM diagnosis. Treatment with CNS-penetrating tyrosine kinase inhibitors (TKIs) resulted in CNS disease control in three of the six cases reported either as single therapy or in combination with radiation therapy (RT). Investigation of differential diagnoses of CBM might be necessary, which include inflammatory and demyelinating disorders, primary brain tumours and infectious diseases, especially neurocysticercosis that might mimic CBM images. Treatment options include RT, CNS-penetrating TKIs and invasive procedures, such as stereotactic drainage. Thus, CBM are associated with ALK-rearranged NSCLC, particularly in patients who use crizotinib and should prompt investigation of differential diagnosis. CNS-penetrating TKIs are effective in the control of solid brain metastases and also seem to be active in CBM as single therapy or in combination with RT.

## Background

A chromosomal inversion leading to the fusion of the anaplastic lymphoma kinase (ALK) gene with the echinoderm microtubule-associated protein-like 4 (EML4) gene was identified in 2007 [1], resulting in the EML4-ALK fusion protein, a therapeutic target in advanced non-small-cell lung cancer (NSCLC). Patients with EML4-ALK fusion and other ALK rearrangements represent 3–7% of all NSCLC cases, usually associated with younger age, never smoking or light smoking history and adenocarcinoma histology (especially in those tumours harbouring signet-ring cell features) [1, 2]. This subset of patients is responsive to the ALK inhibitor crizotinib, with an objective response rate of approximately 60% and a median progression-free survival (PFS) of 8–10 months [3, 4].

However, the majority of the patients who respond to crizotinib will relapse within a few months interval [5, 6], with a median PFS of 10.9 months as a first line therapy and 7.7 months when used after disease progression to chemotherapy [4, 7]. One of the most common sites of progressive disease (PD) in patients with ALK-rearranged NSCLC is central nervous system (CNS), presumably due to the poor blood-brain barrier penetration of crizotinib and the development of mutations related to resistance to crizotinib [5, 8, 9]. The second- and third-generation ALK TKIs have better CNS penetration, showing efficacy in controlling brain metastases in phase-I and phase-II studies [10–13]. Cystic brain metastases (CBM) are a particular variant that have been reported in association with ALK-rearranged NSCLC [14–16] and may represent a diagnostic and therapeutic challenge in this clinical setting. We herein report a case of CBM in ALK-rearranged NSCLC and a literature review.

## Illustrative case

**Figure 1.**
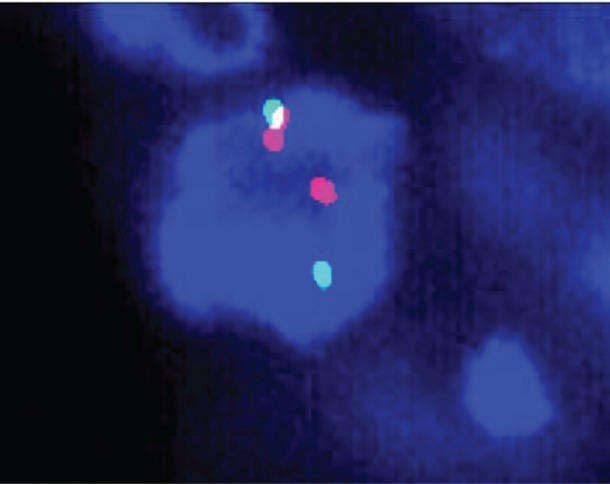
Break-apart fluorescence in situ hybridisation of the presented case.

**Figure 2.**
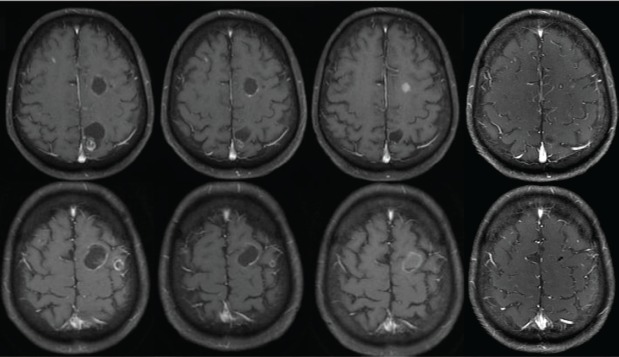
MRI in axial T1-weighted post contrast images demonstrates cystic lesions with ring enhancement in the baseline examination (A and B), delineating cystic areas and ill-defined nodular areas within it, with no surrounding oedema. There was a slight reduction of the lesions after 3 months of ceritinib (C and D), with signs of a more evident response after 6 months (E and F), with reduction of the cystic component of some lesions, and after 9 months (G and H), with reduction of the cystic components and the enhancement.

A 63-year-old non-smoker man was diagnosed with lung adenocarcinoma in 2013, with pleural involvement by the time of the diagnosis (stage IV). The patient was treated with chemotherapy with carboplatin Area under the curve 6 and paclitaxel 200 mg/m^2^ for four cycles every 21 days, with stable disease (SD) as best response to the first line therapy. Follow-up evaluation detected disease progression after 3 months, at the end of platinum-doublet, with lymph node enlargement and pleural effusion. The patient underwent pleurodesis and second line chemotherapy with docetaxel 75 mg/m^2^ every 21 days for eight cycles (6 months). During second-line therapy, the biopsied tumour tissue was submitted to fluorescent *in situ* hybridization analysis (LSI ALK Dual Color Breakapart DNA probe; Vysis®, Abbott Park, IL, U.S.A) that revealed the presence of ALK rearrangement (Figure 1), leading to the initiation of crizotinib 250 mg twice daily. 13 months later, the patient presented with symptoms suggestive of absence crises. A brain magnetic resonance imaging (MRI) revealed brain lesions, suggestive of secondary brain metastases. Chest computed tomography (CT) also showed systemic disease progression in the lung and lymph nodes. Crizotinib was discontinued and the patient was submitted to whole brain radiotherapy (WBRT) (3000 cGy in 10 fractions). Two months after the end of radiotherapy, the patient presented to the emergency department reporting unsteady gait and abnormal mental status characterised by confusion and disorientation. A new MRI of the head demonstrated enlargement of lesions in both cerebral hemispheres, with a predominantly cystic pattern and a contrast enhancement of the cysts wall, without significant surrounding vasogenic oedema. The MRI report suggested that this pattern of lesion was consistent with either neurocysticercosis or metastatic CNS involvement. The patient underwent investigation for neurocysticercosis with cerebrospinal fluid enzyme linked immunosorbent assay, fundoscopic examination and western blot, all of which resulted negative. A biopsy of a new pleural lesion was performed and pathologic analysis demonstrated adenocarcinoma infiltration. After extensive investigation, the cystic brain lesions were attributed to brain metastasis and ceritinib (formerly known as LDK378, Novartis, Basel, Switzerland) was initiated (compassive use). The patient presented important clinical benefit afterwards, with improvement of all neurologic symptoms within 2 weeks. The brain MRI and the chest CT showed partial response (PR) in both brain and systemic lesions (Figure 2).

## Methods

A search in the PubMed/Medline/Lilacs/Scielo database was conducted in order to track relevant papers about CBM in ALK-rearranged NSCLC. The keywords included in the search were ‘cystic brain metastasis’, ‘ALK’, ‘brain metastasis’, ‘crizotinib’ and ‘pathogenesis’. Case reports, series, retrospective, and prospective studies were all eligible for our analysis. Articles were screened and critically analysed.

## Results

Three case reports have been published describing six ALK-rearranged NSCLC patients presenting with CBM [14–16], all of which were under treatment with crizotinib by the time of CBM diagnosis. The treatment and the evolution of these patients are described in Table 1.

Treatment with CNS-penetrating tyrosine kinase inhibitors (TKIs) resulted in CNS disease control in three of the six cases reported: one received ceritinib, presenting intracranial PR (case 3) [15], and two received brigatinib, presenting intracranial PR and SD (cases 4 and 5) [16]. It is noteworthy that the patient who presented PR to brigatinib was not exposed to radiation therapy (RT). Similarly, our case also presented intracranial PR after treatment with ceritinib (Figure 1).

Alongside with CNS-penetrating TKIs, RT was the mainsteam of CBM treatment. In our case report, the patient presented PD 2 months after the end of WBRT. Variable responses to RT were described in four of the previously reported cases who were treated with WBRT and/or stereotactic radiosurgery (SRS). Favourable results were seen in one patient with ALK-rearranged signet-ring cell carcinoma of the lung, who presented complete response after WBRT and crizotinib beyond progression (BP) (case 1) [14] and in another patient who had SD after SRS as monotherapy (case 6) [16]. In the other two cases, however, SRS in association with crizotinib BP (case 3) [15] resulted in PD, even when preceded by WBRT (case 5) [16]. In both cases, CNS disease control was only achieved when CNS-penetrating TKIs (ceritinib and brigatinib, respectively) were initiated [15, 16].

Invasive treatment with cyst drainage using an Ommaya reservoir has also been reported in association with crizotinib BP, resulting in SD in one patient (case 2) [14].

CBM have also been reported in cases of lung adenocarcinoma in which the ALK status was not evaluated. These cases are described in Table 2.

## Discussion

Little is known about the mechanism that leads to the formation of the CBM.

Cumings *et al* [17] evaluating the pathophysiology of any CBM suggested that it occurs because of the tumour degeneration followed by transudation of fluid nearby blood vessels. Another mechanism, suggested by Gardner *et al* [18], was that the alteration of lymphatic drainage leads to interstitial fluid accumulation.

In the case of CBM in ALK-rearranged NSCLC, as mentioned before, all the cases reported to date occurred during treatment with crizotinib. Hayashi *et al* [14] hypothesised that the cystic brain lesions in ALK-rearranged NSCLC might occur because of the action of crizotinib in brain metastases with a cystic degeneration occurring as a response to the target agent. Indeed, crizotinib has shown CNS activity in retrospective analyses, with higher intracranial disease control rates among patients with previously treated brain metastases [19]. However, although some intracranial response to crizotinib occurs [19, 20], this drug has low blood-brain barrier penetration leading to limited CNS response rates [8]. Considering this, one would not expect that an extraordinary response to crizotinib would be the reason for the cystic metastasis. Moreover, when the CNS-penetrating TKIs were used in the reported cases, it resulted in response with reduction and/or resolution of the cystic lesions.

Another explanation for the CBM pointed by Hayashi *et al* [14] is that the signet-ring cells produce abundant mucus resulting in the cyst formation. In fact, in cases submitted to excisional biopsy or cyst drainage, abundant mucinous material was observed [16].

Narayanan *et al* [16] combined these two hypotheses suggesting that the CBM occurred because of a combination of both a histology related to mucus production and an action of crizotinib affecting local disease behaviour in CNS.

It is known that the CNS action of crizotinib is limited because of the low blood-brain barrier penetration of the drug, that achieves a cerebrospinal fluid to plasma ratio of only 0.0026 [8]. Nevertheless, a retrospective analysis of PROFILE 1005 and PROFILE 1007 trials has shown intracranial response rates of 18% in patients without prior brain radiotherapy and 33% in patients with prior brain radiotherapy [19]. When compared with chemotherapy, crizotinib also showed superior intracranial disease control rates (disease control rate of 85% with crizotinib versus 45% with chemotherapy in 12 weeks and 56% and 25%, respectively, in 24 weeks) [20]. Therefore, there seems to exist some action of crizotinib in CNS. This limited action could justify the formation of the CBM. While part of the tumoural cells would suffer necrosis with crizotinib action, a considerable part of the cells would remain viable, leading to the fluid formation surrounded by active tumoural cells in the cyst wall.

Despite the exposed hypothesis, it still remains to be clarified if crizotinib is in fact related to the development of the CBM or if it occurs regardless of crizotinib use in ALK-rearranged NSCLC.

One possibility is that the CBM would not occur because of a direct effect of crizotinib, but actually because of the combination of two factors: the actuation of the CNS as a sanctuary during crizotinib use associated with the longer life expectation of these patients in use of anti-ALK TKIs. Because of this, after 2 years of crizotinib use, 50–60% of the patients present brain metastases [21]. This explanation might justify as well the higher development of the CBM as a variant pattern.

## Differential diagnoses

Despite the increasing number of CBM reported in patients with ALK-rearranged NSCLC, this presentation remains unusual. Thus, it might be necessary to consider some differential diagnoses, which might present with a similar radiographic pattern. Table 3 summarizes the possible differential diagnoses of cystic brain lesions.

Brain metastasis is the most common intracranial tumour in adults [22], occurring in up to 30% of patients with cancer [23]. Lung cancer is the leading cause of brain metastasis, followed by melanoma and renal cell carcinoma [23]. In ALK-rearranged NSCLC, lifetime incidence of brain metastases approaches 50% [24]. The classical radiological finding of a brain metastasis is a solid and/or heterogeneous ring-enhancing mass with well-defined margins and extensive surrounding oedema [22], commonly found in the grey-white junction. CBM, however, usually show no vasogenic oedema and mild or no contrast enhancement, limited to the cyst wall [16].

Neurocysticercosis is an endemic disease in developing countries of Africa, Asia, Central and South America [25] and is the most common parasitic disease of the brain [26]. In some situations, brain images are unable to accurately differentiate neurocysticercosis from CBM. There are some cases reported in which this differentiation was needed [27–32]. Each of the four developmental stages of the cysts determines different imaging findings: (1) vesicular stage is the earliest phase, characterised by thin-walled cysts, often with the classical mural nodule representing the scolex and usually with neither oedema nor contrast enhancement [22, 33, 34]; (2) colloidal-vesicular stage marks the beginning of cyst degeneration which leads to host inflammatory response and consequent pericystic oedema and cyst wall enhancement [34]; (3) granular nodular (or healing) stage imaging pattern on CT scan consists of isoattenuated cysts with hyperattenuated calcified scolex, with persistent contrast enhancement and surrounding oedema [22] and (4) quiescent (residual) stage radiological pattern is represented by calcified nodules without mass effect [33, 34]. Despite imaging studies and serological tests, which are the basis of neurocysticercosis diagnosis, some cases might still require invasive procedures, such as stereotactic brain biopsy, to establish the correct diagnosis [23].

## Management

There is little evidence available to suggest a standard treatment of the CBM in ALK-rearranged NSCLC.

Based on the case reports published to date, treatment options for the CBM include RT (WBRT or SRS) and/or CNS-penetrating TKIs.

Since ALK-rearranged NSCLC usually occurs in younger patients, many of whom are, otherwise, healthy and functional, the risk of cognitive sequel related to WBRT is an important issue when considering RT. Therefore, CNS-penetrating TKIs should be preferred over WBRT as first line therapy in this clinical setting. SRS, on the other hand, is related to less cognitive sequel and it is the preferred RT, when feasible [35–37].

In the cases treated with RT, continuation of crizotinib BP might also be considered. In the case of oligoprogressive disease, when it is considered that the patient is still presenting clinical benefit with crizotinib, the continuation of the drug might extend its benefit [38].

To date, CNS-penetrating TKIs, including ceritinib and alectinib, are approved for use after progression to crizotinib [10–13]. Two recently published phase-III trials showed benefit of ceritinib (ASCEND-4) [39] and alectinib (ALEX [40] and J-ALEX) [41] in first-line scenario. In the phase-II and -III trials, these drugs presented intracranial responses rates ranging from 39% to 75% [10–13, 39]. Including patients with no prior RT had intracranial response rate as high as 69.2% with ceritinib [39] and 67% with alectinib [12]. The third-generation anti-ALK TKIs brigatinib and lorlatinib also have shown CNS efficacy in earlier phase studies, with intracranial response rate of 41% and 44%, respectively [42, 43].

Based on the case reports of CBM, it seems that the CNS-penetrating TKIs might also have efficacy in the CBM as well as they have in the solid brain lesions. One of the reported cases presented PR to a CNS-penetrating TKI without prior brain irradiation [16], a strategy that might even allow avoidance or postponement of brain radiotherapy.

Another possible strategy described for the treatment of CBM is stereotactic drainage. Kim *et al* [44] suggested that for large CBM, regardless of the primary tumour site, the combination of stereotactic drainage and radiosurgery might be considered. Since the large size of the cysts might limit RT, cyst drainage would allow reducing the tumour volume, turning it possible to perform radiosurgery. Wang *et al* [45] evaluated the treatment of large CBM with stereotactic aspiration followed by Gamma Knife radiosurgery (GKRS). 48 patients were analysed, of which 60% had NSCLC as the primary tumour. Tumour control rate in 3 months was 91.7%. Franzin *et al* [46] reported similar results with a local tumour control rate of 91.3% in 3 months after the treatment of CBM with stereotactic drainage and GKRS in 23 patients (63% of which had NSCLC as the primary tumour). This strategy might be considered for large CBM.

## Conclusions

Based on the present and on the previous case reports, we conclude that ALK-rearranged NSCLC is associated with CBM, particularly in patients in use of crizotinib. Despite that, this metastatic pattern is still unusual and differential diagnosis should be investigated when the etiologic diagnosis of the cystic lesion is not clear.

Regarding the treatment options, the CNS-penetrating TKIs are effective in the control of solid brain metastases and also seem to be active in the CBM. RT (WBRT or SRS), with or without continuation of the TKI, might also be considered, with variable results. Moreover, stereotactic drainage might be considered previous to the RT for large CBM.

With the increasing experience with the management of ALK-rearranged NSCLC, it is expected that more cases will be studied, leading to improved knowledge about the best strategy for treating these patients with CBM.

## Conflicts of interest

Guilherme Nader Marta, Renata R da C Colombo Bonadio and Henrique Bortot Zuppani have no conflicts of interest to declare.

Renata Eiras Martins—travel grants: Pfizer; lectures: Pfizer; investigator of sponsored trials: Pfizer, Novartis, Roche.

Gilberto de Castro Junior—member of advisory board: Pfizer, Novartis; travel grants: Pfizer, Novartis; lectures: Pfizer; investigator of sponsored trials: Pfizer, Novartis, Roche.

## Authors’ contributions

- **Conception and design of study:** Guilherme Nader Marta, Renata Eiras Martins
- **Acquisition of data:** Guilherme Nader Marta, Renata R da C Colombo Bonadio, Renata Eiras Martins, Henrique Bortot Zuppani, Gilberto de Castro Junior
- **Analysis and/or interpretation of data:** Guilherme Nader Marta, Renata R da C Colombo Bonadio, Renata Eiras Martins, Gilberto de Castro Junior
- **Drafting the manuscript:** Guilherme Nader Marta, Renata R da C Colombo Bonadio
- **Revising the manuscript critically for important intellectual content:** Renata Eiras Martins, Henrique Bortot Zuppani, Gilberto de Castro Junior

## Figures and Tables

**Table 1.** Case reports of CBM in ALK-rearranged NSCLC.

Article	Case	Neoplasia	Previous crizotinib	Smoking status	Treatment	Response
Hayaski *et al* [47]	Case 1	Signet-ring cell carcinoma of lung	Yes	Former smoker	WBRT + crizotinib BP	IC CR
	Case 2	Lung acinar adenocarcinoma	Yes	Non-smoker	Ommaya reservoir to drain the cystic mass + crizotinib BP	IC SD
Saraceni *et al* [15]	Case 3	Lung adenocarcinoma	Yes	Non-smoker	Crizotinib BP + Cyberknife therapy; after CNS (central nervous system) progression, initiated ceritinib	IC PR to ceritinib
Narayan *et al* [16]	Case 4	Lung adenocarcinoma	Yes	Former light smoker	Brigatinib	IC PR
	Case 5	Mucinous adenocarcinoma	Yes	Non-smoker	WBRT + crizotinib BP; followed by SRS; followed by brigatinib	IC SD with brigatinib
	Case 6	NSCLC (not specified)	Yes	Former smoker	SRS	IC SD

CRZ: crizotinib; WBRT: whole brain radiation therapy; BP: beyond progression; SRS: stereotactic radiosurgery; IC CR: intracranial complete response; IC PR: intracranial parcial response; IC PD: intracrainial progressive disease; IC SD: intracranial stable disease

**Table 2.** Case reports of CBM in NSCLC without ALK status report.

Article	Case	Neoplasia	Smoking status	Treatment	Evolution
Surov *et al* [48]	Case 1	Lung adenocarcinoma	NR	NR	NR
Mota *et al* [28]	Case 1	Lung adenocarcinoma	Smoker	WBRT	IC PR
Costa *et al* [23]	Case 1	Lung adenocarcinoma	Non smoker	Systemic chemotherapy with carboplatin and paclitaxel	IC SD

NR: not reported; WBRT: whole brain radiation therapy; IC CR: intracranial partial response; IC CR: intracranial stable disease

**Table 3.** Differential diagnoses of brain cystic lesions.

Bacterial	Fungal	Parasitic	Inflammatory and demyelinating disorders	Neoplastic
Pyogenic abscess	Cryptococcosis	Neurocysticercosis	Multiple sclerosis	Metastases
Tuberculous abscess	Actinomycosis	Amoebic abscess	Acute disseminated encephalomyelitis	Primary brain tumour
MAC	Rhodococcosis	Toxoplasmosis	Sarcoidosis	
Listeriosis	Zygormycosis	Chagas disease	Vasculitis	
	Coccidiomycosis		Behcet disease	
	Paracoccidiomycosis			
	Mucormycosis			
	Aspergillosis			
	Nocardiois			
	Histoplasmosis			

MAC: mycobacterium avium intracellulare
